# Facial and Prosodic Emotion Recognition Deficits Associate with Specific Clusters of Psychotic Symptoms in Schizophrenia

**DOI:** 10.1371/journal.pone.0066571

**Published:** 2013-06-20

**Authors:** Huai-Hsuan Tseng, Sue-Huei Chen, Chih-Min Liu, Oliver Howes, Yu-Lien Huang, Ming H. Hsieh, Chen-Chung Liu, Jia-Chi Shan, Yi-Ting Lin, Hai-Gwo Hwu

**Affiliations:** 1 Department of Psychosis Studies, Institute of Psychiatry, King’s College London, London, United Kingdom; 2 Department of Psychiatry, National Taiwan University Hospital and College of Medicine, National Taiwan University, Taipei, Taiwan; 3 Department of Psychology, National Taiwan University, Taipei, Taiwan; 4 Department of Psychiatry, Cathay General Hospital, Taipei, Taiwan; 5 Department of Psychiatry, National Taiwan University Hospital Yun-Lin Branch, Dou-Liou City, Yun-Lin, Taiwan; University of Groningen, The Netherlands

## Abstract

**Background:**

Patients with schizophrenia perform significantly worse on emotion recognition tasks than healthy participants across several sensory modalities. Emotion recognition abilities are correlated with the severity of clinical symptoms, particularly negative symptoms. However, the relationships between specific deficits of emotion recognition across sensory modalities and the presentation of psychotic symptoms remain unclear. The current study aims to explore how emotion recognition ability across modalities and neurocognitive function correlate with clusters of psychotic symptoms in patients with schizophrenia.

**Methods:**

111 participants who met the DSM-IV diagnostic criteria for schizophrenia and 70 healthy participants performed on a dual-modality emotion recognition task, the Diagnostic Analysis of Nonverbal Accuracy 2-Taiwan version (DANVA-2-TW), and selected subscales of WAIS-III. Of all, 92 patients received neurocognitive evaluations, including CPT and WCST. These patients also received the PANSS for clinical evaluation of symptomatology.

**Results:**

The emotion recognition ability of patients with schizophrenia was significantly worse than healthy participants in both facial and vocal modalities, particularly fearful emotion. An inverse correlation was noted between PANSS total score and recognition accuracy for happy emotion. The difficulty of happy emotion recognition and earlier age of onset, together with the perseveration error in WCST predicted total PANSS score. Furthermore, accuracy of happy emotion and the age of onset were the only two significant predictors of delusion/hallucination. All the associations with happy emotion recognition primarily concerned happy prosody.

**Discussion:**

Deficits in emotional processing in specific categories, i.e. in happy emotion, together with deficit in executive function, may reflect dysfunction of brain systems underlying severity of psychotic symptoms, in particular the positive dimension.

## Introduction

Difficulty in emotional processing is one of the core deficits in patients with schizophrenia [1], [2]. They perform significantly worse on emotional recognition tasks than healthy participants, across a range of sensory modalities (See review of Edwards et al. [3]). Rather than a generalized deficit in emotion recognition, there appear to be specific deficits in patients with schizophrenia, including being less accurate in recognizing negative emotions than positive ones, especially the fearful and sad emotions across facial expression [4], [5], [6] and prosodic emotion recogntion [7]. Emotion perception and recognition is the initial stage of emotional processing, and subsequently, the basis of social cognition. As such defects in recognizing emotion may contribute to patients’ poor interpersonal and social functioning [8], [9].

Existing literature demonstrates associations between emotional processing difficulties and the severity of clinical symptoms in schizophrenia. A positive association between nonverbal emotion recognition deficits with severity of negative symptoms [10], [11], [12] has been confirmed by a meta-analysis [13] in facial modality. Although less confirmative, it is evidenced that both facial [14], [15], [16] and prosodic modalities are associated with the positive symptoms of schizophrenia [17]. Nevertheless, such associations may not be as stable over time as those with negative symptoms [14], implying that the association may be more state-dependant.

Whilst fewer studies of non-verbal emotion recognition have addressed the relationship between emotion-specific deficits and the presentation of psychotic symptoms in different domains, there indeed exist some evidences of specificity. For example, negative emotion recognition difficulties, especially fear, is related to negative symptoms [7], [18]. Furthermore, positive emotion recognition difficulties have also been reported to be associated with positive and cognitive symptom domains [15]. Since each emotion conveys distinct, or even opposite, social information, differential connections between emotion-specific deficits and separate symptom dimensions can be expected and needs further exploration.

In real life non-verbal emotional information is usually simultaneously presented across modalities. A concurrent assessment across sensory modalities may provide further information regarding the ability of emotional processing [19], while minimizing the confounding effects within each single modality. But to date, studies concurrently examining relationships between emotional processing deficits across sensory modalities and symptomatology in schizophrenia are scarce. Existing evidence suggests that emotion recognition across facial and prosodic modalities is negatively associated with negative and cognitive symptom dimensions [7], [20]. However, the research paradigms for visual and auditory sensory modalities were unmatched in these studies. A paradigm with parallel visual and auditory emotional conditions will be suitable for measuring nonverbal emotion recognition across sensory modalities.

Examining emotion processing deficits across sensory modalities does not eliminate the possibility that modality-specific emotional processing deficits also contribute to the clinical presentation of psychotic symptoms. While facial emotion recognition difficulties are often associated with negative symptoms in schizophrenia, prosodic emotion recognition has been reported to associate with cognitive symptoms [20], [21]. Furthermore, a link between prosodic emotional processing difficulties and the presence of hallucinations or delusions has been demonstrated [17], [22]. More specifically, patients with these positive symptoms seem to have difficulties correctly identifying happy emotion, possibly because of a tendency to attribute fear or sadness to any stimulus [23]. Thus further comparison of the correlation between clinical symptoms and facial and prosodic emotion recognition is essential to elucidate the relationship.

Deficits in executive function and sustained attention are two well-studied neurocognitive deficits in patients with schizophrenia, which are robustly linked to the genetic predisposition for schizophrenia [24]. Studies suggest emotional recognition tasks, especially those using forced choices, are related to executive function [25] and sustained attention [26], indicating the deficit may at least partially be explained by these neurocognitive deficits. Furthermore, negative symptoms were associated with worse neurocognitive reserve, clinical course, and social functioning [27]. Hence, to investigate the contribution of nonverbal emotion recognition deficits to severity of individual symptom domains, it is important to delineate the contribution of these two neurocognitive functions while using a force choice paradigm.

As stated above, both nonverbal emotion recognition ability and neurocognitive function have been shown significantly related to clinical symptom severity, but to what extent nonverbal emotional recognition ability across modalities and neurocognitive functions contribute to the severity of different symptom clusters remain unclear. Moreover, emotion-specific and modality-specific contribution may also exist. A careful examination of these interrelationships will elucidate the role of nonverbal emotional processing deficits in the composition of psychopathology of schizophrenia. By using a culture-suitable nonverbal instrument, the current study aims to explore the relationships among the severity of psychotic symptoms, nonverbal emotion recognition ability, and neurocognitive function in patients with schizophrenia, and delineate the contribution of emotional processing difficulties from the neurocognitive deficits and other clinical variables to different symptom clusters. The emotion-specific and modality-specific contributions to individual symptom clusters will then be examined.

We postulate that impairment of nonverbal emotion recognition ability across different sensory modalities contributes to symptom severity in schizophrenia, in particular negative and cognitive symptoms, and neurocognitive dysfunctions can at least explain part of this contribution. We also postulate that after accounted for neurocognitive deficits, nonverbal emotional recognition deficits across modalities may still predict severity of certain symptom dimensions, such as positive or behavioral symptoms.

## Methods

The institutional review board of the National Taiwan University Hospital approved the study.

### Participants

#### Patients

One hundred and eleven patients who met the DSM-IV diagnostic criteria for schizophrenia were recruited from outpatient clinics or rehabilitative daycare services in National Taiwan University Hospital from October 2006 to September 2008. All participants gave fully informed written consent to participate. Each participant’s capacity to consent was evaluated by the psychiatrist who made the referral to the study. When the capacity to consent was suspected to be reduced, consent from another family member was required in addition to the participant’s consent. All potential participants who declined to participate or otherwise did not participate were not disadvantaged in any way by not participating in the study.

The diagnoses were confirmed by consensus among two board-certified psychiatrists using available clinical information from medical and psychiatric records. All patients received semi-structured Diagnostic Interview for Genetic Study (DIGS) – Chinese Version [28], and those with a diagnosis other than schizophrenia and those with a history of severe physical illness, neurological abnormality such as severe visual and hearing impairment, or substance abuse were excluded. Sample characteristics are presented in tables 1 and 2, and information of medication in table 3.

**Table 1:** Demographic background and cognitive capacity of patients with schizophrenia and healthy participants.

	Schizophrenia (*n* = 111)Mean ± SD	Control (*n* = 70) Mean ± SD	*p* value
**Demographic variables**			
Gender – no.	M/F :51/60	M/F : 29/41	0.36
Current age – years	38.23±10.13	36.26±11.34	1.21
Education – years	15.89±3.43	13.45±10.0	− 5.38***
**Cognitive capacity**			
Estimated VIQ	94.58±16.91	109.97±15.34	− 6.87***
Estimated PIQ	89.55±19.32	112.30±16.23	− 8.19***
Estimated FIQ	92.48±16.33	109.55±18.34	− 8.51***
Working Memory Index (WMI)	96.11±13.87	109.88±14.80	− 7.61***
**Clinical information**			
Age of Onset	24.81±8.18	–	–
Duration of illness	13.84±9.74	–	–
**Neurocognitive ability (in Z scores)**			
	**Schizophrenia (n = 92/111)**	–	–
WCST Total errors	0.60±1.21	–	–
WCST perseveration errors	0.74±1.44	–	–
WCST conceptual responses	−0.57±1.16	–	–
CPT d’	−0.71±1.29	–	–
CPT beta	−0.32±1.57	–	–

*** *p*<0.001; using Independent t test for continuous variables; Pearson’s chi-square test for categorical variables.

**Table 2:** Clinical symptom ratings in patients with schizophrenia by PANSS and dimension scores.

PANSS total scores, scale scores and dimensional scores in patients with schizophrenia (n = 92/111)	
Total PANSS Scores	55.60±15.66
Positive Scale	11.91±4.43
Negative Scale	14.67±5.85
General Scale	25.33±7.88
Supplement Scale	3.69±1.39
**Symptom Dimension Score**	
Delusion/Hallucination	8.58±4.52
Negative Symptoms	17.37±6.54
Disorganized thought	11.64±4.11
Hostility/excitement	6.40±2.73

**Table 3:** Current medication in Patients with Schizophrenia (*n* = 111).

Medication Name	Number of patients	Range of dosage (mg)	Average dosage (mg)	
**Antipsychotics**				
Amisulpride	9	200–1200	522.22	per day
Aripiprazole	6	7.5–30	17.50	per day
Chlorpromazine	2	50–100	75.00	per day
Clozapine	21	75–500	292.50	per day
Flupentixol	1	5–5	5.00	per day
Haloperidol	7	5–20	12.14	per day
Olanzapine	18	2.5–30	15.42	per day
Quetiapine	7	25–700	360.71	per day
Risperidone	22	1–9	3.82	per day
Sulpiride	16	200–1000	440.63	per day
Trifluoperazine	1	30	30.00	per day
Ziprasidone	3	80–160	133.33	per day
Zotepine	6	25–300	195.83	per day
**Long Acting Antipsychotics**				
Fluphenazine decanoate	2	6.25–12.5	9.38	per week
Flupentixol	4	5–20	10.83	per week
Risperidone	4	5–12.5	10.63	per week
**Antidepressants**				
Bupropion	1	150–150	150.00	per day
Fluoxetine	6	20–60	31.67	per day
Fluvoxamine	3	50–100	83.33	per day
Mirtazapine	1	30	30.00	per day
Moclobemide	1	150	150.00	per day
Sertraline	5	50–50	50.00	per day
Trazodone	2	50–50	50.00	per day
Venlafaxine	2	150–150	150.00	per day
**Mood stabilizers**				
Valproic Acid	4	500–1000	750.00	per day

All participants received the nonverbal emotion recognition task, Diagnostic Analysis of Nonverbal Accuracy-2 Taiwan version (DANVA-2-TW) [29]. Ninety-two of them agreed to receive clinical interview of symptom severity, the Positive and Negative Symptom Scale, Mandarin version (PANSS-M) [30] and completed the whole neurocognitive assessment schedule.

#### Healthy participants

Seventy age- and gender-matched community volunteers and hospital staffs without a lifetime or current history of psychiatric disorder based on the information collected using the DIGS – Chinese Version were recruited as healthy participants. All participants gave fully informed written consent to participate. All of them received DANVA-2-TW and general intellectual assessment.

### Measurements

#### Non-verbal emotion recognition (DANVA2-TW)

Studies suggest that race, culture, gender, and psychiatric illness may considerably affect the accuracy of judging nonverbal emotions [32], [33], [34]. A culturally suitable nonverbal measure for Han Chinese, the Diagnostic Analysis of Non-verbal Accuracy 2-Taiwan version (DANVA-2-TW) has been selected for measurement of emotion recognition accuracy. The DANVA-2-TW is a parallel version of the Diagnostic Analysis of Non-verbal Accuracy 2 [35] which has been used widely in both non-clinical and clinical research, including schizotypal personality disorder [36]. The DANVA-2-TW is a computerized measure, comprises 60 facial photographs and 60 voice clips representing specific emotions and intensities, including twelve for each of the four (happy, sad, angry, fearful) basic emotion categories and twelve neutral stimuli [29]. Different from its original one, DANVA 2-TW consists of the neutral stimuli that were designed as additional measures for forced interpretation biases for ambiguous emotional stimuli. In the present study, neutral stimuli were not included in analysis.

The facial photos were displayed on a computer screen with a full screen size and a resolution of 1024×768, and voice clips were delivered to the participants through an earphone. The faces and voices were presented in different sessions, and the participants were asked to make a forced choice among the four emotional categories. The accuracy values were ratios of correctly answered items within per emotion categories, ranged from 0 (completely inaccuracy) to 1 (complete accuracy). The overall accuracy of facial or prosodic emotion recognition was the average of all emotions of the same modality. The total accuracy of nonverbal emotion recognition was derived from averaging overall facial and overall prosodic accuracy. Both subtests were reported to have satisfactory inter-rater and the test-retest reliability [29], [37].

#### Positive and negative symptom scale (PANSS) [31]

The PANSS has 33 items rated on a 7-point scale based on a semi-structured interview. A four-dimension structure has been extracted from the mandarin version of PANSS, PANSS-M, based on 163 Han Chinese patients with schizophrenia in Taiwan [27], including Delusion/Hallucination (P1,P3,P6,G9); Negative Symptoms (N1,N2,N3,N4,N5,N6,G7,G10,G13); Disorganized thought (P2,N7,G11,G15); Hostility/excitement (P7,G4,G8,G14,G16,S1,S2,S3,S4). The four-dimension structure includes 26 items out of 33 items of the original PANSS-M.

### Clinical and Neurocognitive Assessment Schedule

The schedule contained information regarding demographic features for all participants, including age, gender, educational level, and clinical features for patients, including age of onset of psychotic symptoms, and duration of illness. The cognitive function assessment included “1–9” version of continuous performance test (CPT), computerized version of Wisconsin card sorting test (WCST), and five subtests from Wechsler Adult Intelligence scale - III.

#### Continuous performance task (CPT)

A CPT machine from Sunrise Systems, v. 2.20 (Pembroke, MA, USA), was used to assess sustained attention. The procedure has been described in detail elsewhere [38].

Briefly, numbers from zero to nine were randomly presented for 50 milliseconds, each at a rate of one per second. Subjects were asked to respond whenever the number nine was preceded by the number one on the screen. Sensitivity (*d*) was derived from the hit rate (probability of a response to target trials) and false-alarm rate (probability of response to non-target trials). CPT performance indicators were calculated as the *z* scores, which were adjusted for demographic features, and were subsequently used for further study analyses.

#### Wisconsin card sorting test (WCST)

We employed a computerized version of the WCST that had been applied in several previous studies (for example, [24], [27]) of the Taiwanese population. Subjects were required to match 128 response cards to the four stimulus cards along one of three dimensions (color, form, number), by pressing one of the four number keys on the computer keyboard. The indexes of WCST used for this study were the following: (1) perseverative errors, which reflected the tendency towards perseveration; and (2) categories achieved, which was the number of times that 10 consecutive correct responses were made, which reflected overall success. These indicators were found to be impaired in schizophrenia probands and in their first-degree relatives [39], [40]. WCST performance indicators were transformed into z scores with adjustment for the demographic features. The indicators were subsequently used for further analyses.

#### Subtests from wechsler adult intelligence scale – III

Five (digit span, block design, arithmetic, digit symbol substitution, and information) subtests modified from Blyler’s short form of the WAIS-III for patients with schizophrenia [41] were employed to assess the potential confounding effect of cognitive capacity. Working Memory Index (WMI) was estimated by Digit Span and Arithmetic.

### Statistical Analyses

The demographic data were compared between groups using independent *t* tests and Chi-square tests for continuous and categorical variables, respectively. The effect of diagnostic group on emotion was assessed using univariate analyses of covariance (ANCOVA) with group as the independent variable and full scale IQ and education as covariates. Correlation analyses using Pearson’s *r* were performed among all emotional recognition and total and dimension scores of PANSS. A conservative significance level of *p*<0.01 was applied for correlation analyses accounting for multiple comparisons. Multiple bidirectional stepwise linear regression analysis using the following stepping method criteria: probability of *F* to enter the model, <0.05; to remove from the model, >0.10 was performed, in which PANSS total score and dimension scores were treated as dependent variables separately, and the emotional recognition indexes, demographic and clinical data and cognitive performance including age, gender, education, duration of illness, age of onset, estimated full scale IQ, and WCST and CPT performance indicators were entered as predictor variables. Results of forward and backward stepwise multiple linear regression analyses were then compared using the same inclusion and elimination criteria.

All statistic analyses were computed using Statistical Package for Social Science (SPSS, Chicago, Illinois, USA) software version 19.0 for Windows. The statistical analyses were considered significant if *p*-values were smaller than 0.05 while not elsewhere specified.

## Results

### Demographic and Clinical Characteristics

There were no significant differences in age and gender between healthy participants and patients with schizophrenia (Table 1). However, patients with schizophrenia were significantly lower in education and all cognitive measures including estimated IQ and working memory index (Table 1). The symptom and medication history of the patients are shown in Table 2 and Table 3 respectively. The mean dosages of various medications were around the recommended dose level.

### Emotion Recognition

Table 4 shows the results of the emotion recognition task by group. Emotion recognition ability of patients with schizophrenia was significantly worse than that of healthy participants, in both face (0.68 and 0.77 respectively, ANCOVA *F = *6.69, *p* = 0.01) and voice channels (0.65 and 0.81 respectively, ANCOVA *F* = 15.78, *p*<0.001). Fearful emotion recognition was the most discriminative emotion between patients with schizophrenia and healthy participants (0.52 vs. 0.70, ANCOVA *F* = 14.74, *p* = 0.001), after controlling for differences in education and verbal IQ.

**Table 4:** ANCOVA for the differences of emotion recognition ability between patients with schizophrenia and healthy participants.

	Schizophrenia (*n* = 111) Mean ± SD	Control (*n* = 70) Mean ± SD	*F* (1, 177)	Effect size	*p* value
**Overall Emotional Recognition Abilities**					
Overall	0.67±0.13	0.79±0.08	14.16	1.11	<0.001***
Facial	0.68±0.13	0.77±0.08	6.69	0.83	0.01*
Voice	0.66±0.16	0.81±0.10	15.78	1.12	<0.001***
**Separate Emotional Categories**					
Happy	0.79±0.15	0.89±0.09	8.97	0.55	0.03*
Sad	0.71±0.18	0.84±0.12	8.00	0.84	0.02*
Angry	0.67±0.15	0.74±0.12	4.87	0.52	0.07+
Fearful	0.52±0.23	0.70±0.16	14.74	0.91	0.001**

+ *p*<0.10;

* *p*<0.05;

** *p*<0.01;

*** *p*<0.001.

Effect size was the standardized mean difference between groups (Cohen’s *d*).

### Correlation Analysis

#### Correlation between demographic, clinical, neurocognitive variables and PANSS scores

This correlation analysis was aimed to explore the most important factors which may influence the clinical symptoms, thus correction for multiple comparison was not applied. In the correlation analysis with demographic, clinical and cognitive data, PANSS total score was negatively and moderately correlated with the perseverative errors (*r* = –0.31, *p*<0.01) in WCST. There was no correlation between PANSS total score and demographic and clinical data. However, while inspecting the dimension scores separately, educational level was negatively and modestly correlated with negative symptom dimension (*r* = −0.23, *p*<0.05). Furthermore, age of onset was negatively and modestly correlated with hostility/excitement dimension (*r* = −0.25, *p*<0.05). All these variables with significant correlation with PANSS total score or dimension scores entered as a potential predictor for further regression analysis in addition to performances in emotional tasks.

#### Correlation between nonverbal emotion recognition and PANSS scores

A conservative threshold of significance, *p*<0.01, was applied considering multiple comparisons have been performed. A trend of negative association was found between the overall nonverbal emotional recognition score and the PANSS total score (*r* = −0.21, *p*<0.05). In terms of different emotions across modalities, the accuracy of recognizing happy emotion, but not the other emotions, was negatively and moderately correlated with the PANSS total score (*r* = −0.33, *p* = 0.001). With regard to the modality across emotions, only the accuracy of voice modality is negatively and modestly correlated with PANSS total score (*r* = −0.25, *p* = 0.01; See Table 5).

**Table 5:** Correlations between emotion recognition and clinical symptoms (*n* = 92).

	Overall	Faces	Prosodic	Happy	Sad	Anger	fear
PANSS	−.21+	−.11	−.25+	−.33*	−.16	−.04	−.13
NegativeSymptoms	−.21+	−.15	−.22*	−.18	−.19+	−.07	−.17
Delusion/Hallucination	−.09	−.05	−.11	−.27*	−.13	.05	.02
DisorganizedThought	−.15	−.05	−.21*	−.27*	−.09	−.05	−.08
Excitement/Hostility	−.12	−.04	−.17	−.17	−.07	.02	−.12

+ *p*<0.05;

* *p*<0.01; using Pearson’s correlation coefficient *r*.

While looking into separate dimensions of the PANSS, a trend of negative association was found between overall accuracy of nonverbal emotion recognition and negative symptom dimensions (*r* = −0.21, *p*<0.05). Regarding different emotion categories across modalities, accuracy of happy emotion was negatively and modestly correlated with Delusion/Hallucination dimension (*r* = −0.27, *p*<0.01) and Hostility/Excitement dimension (*r* = −0.27, *p*<0.01; See Table 5).

### Regression Analysis

Age, gender, education, duration of illness, age of onset, estimated full scale IQ, and WCST and CPT performance indicators as well as indices of emotion recognition accuracy were entered as predictors. Forward and backward stepwise multiple linear regression analyses were compared with bidirectional stepwise multiple linear regression analyses and led to the same results.

#### PANSS total score as dependent variable

When overall accuracy of nonverbal emotion recognition was entered as one of the predictor variables, perseveration error in WCST was the only significant predictor of PANSS total score (*p*<0.01, *R* ^2^ = 0.08).

When emotion categories were considered separately as one of the predictor variables, the accuracy of happy emotion recognition and age of onset became another two significant predictors in addition to the perseverative errors in WCST (*p*<0.01, *p*<0.05 and *p*<0.05 respectively, change of *R* ^2^ = 0.11, 0.05 and 0.06, respectively; overall *R* ^2^ = 0.22).

When nonverbal emotion recognition in different modalities were considered separately as the predictor variables, the perseveration error in WCST remained significant. Age of onset and accuracy of voice modality significantly predicting PANSS total score (*p* = 0.05 and 0.05, respectively). No significant findings were resulted for facial emotions.

#### PANSS dimension scores as dependent variables

Considering different dimensions of the PANSS, recognition of happy emotion and age of onset were the only two significant predictors of delusion/hallucination dimension (*p*<0.01, change of *R* ^2^ = 0.08 and *p*<0.05, change of *R^2^* = 0.04, respectively) and disorganized thoughts dimension (*p*<0.01, change of *R* ^2^ = 0.10 and *p*<0.05, change of *R^2^* = 0.04, respectively). To further elucidate the possibility of modality-specific prediction of happy emotion for symptom clusters, emotion recognition in happy faces and happy voices were then considered separately as predictor variables. Recognition of happy voices remained significant to predict both delusion/hallucination dimension (*p*<0.05, change of *R* ^2^ = 0.05) and disorganized thoughts dimension (*p*<0.001, change of *R^2^* = 0.13). No significant findings were noted for recognition of happy faces.

On the other hand, the perseverative responses error of WCST was the only predictor of negative symptom dimension (p<0.05, *R^2^* = 0.07). Furthermore, together with age of onset, the perseverative errors of WCST predicted the excitement/hostility dimension of PANSS (*p*<0.05 and *p*<0.05, respectively; change of *R^2^* = 0.07 and 0.05, respectively).

## Discussion

To our knowledge, this is the first study attempting to use the accuracy of separate emotion category and modality to predict concurrent symptom clusters of schizophrenia. The study evidenced nonverbal emotion recognition deficits across modalities, and these deficits had emotional-specific and modality-specific correlations with separate symptom dimensions after controlling for demographic characteristics and neurocognitive functions. More noteworthy, the present study found that, in patients with schizophrenia relative to healthy participants; their voice modality was more prominent than facial modality; and greater impairment in distinguishing fear emotions. The findings are comparable with our previous study [19] and some other studies [3].

The most salient finding of the present study is that the nonverbal emotion recognition accuracy across modalities and neurocognitive functions predicted severities of different symptom dimensions of schizophrenia. Together with age of onset, the accuracy of happy emotion recognition significantly predicted scores of delusion/hallucination and disorganized thoughts dimension; in contrast, the performance in WCST predicted the scores of negative symptom dimension and excitement/hostility dimension. The result was in line with our hypothesis, and suggests in addition to neurocognitive function which has been profoundly investigated, a differential contribution of nonverbal emotional processing dysfunction to clinical symptoms of schizophrenia.

Hence we agree with Larøi et al. [15] that nonverbal emotion recognition deficit aggregates with specific symptom dimension instead of global symptoms in schizophrenia. In current study, the aggregation seems to be emotion-specific across facial and prosodic modalities. More specifically, lower accuracy of recognizing happy emotion was associated with higher levels of positive symptoms and disorganized thoughts. The findings are in line with previous studies, suggesting patients with higher positive symptoms have difficulties correctly identifying happy facial expressions [15], [23] and prosodic voices [17]. The correlation between nonverbal emotion recognition and cognitive symptoms (disorganized thought dimension in present study) has also been reported in both modalities, especially prosodic one [20], [21]. As the easiest emotion to identify, failure of recognizing happy emotion might reflect the presence of cognitive symptoms.

Emotion recognition for prosodic modality was more predictive of above-mentioned specific symptom dimensions. In line with Shea et al. [17], our further exploration of modality effects within happy emotion demonstrated that scores of recognizing prosodic voice was more predictive for delusion/hallucination and disorganized thought. The result is partially in line with studies showing correlations between vocal emotion identification across emotions and cognitive subscale, but not with either positive or negative factor scores [20], [21]. Together with our findings, the absence of significant correlation with positive symptoms after averaging across emotions in these studies suggests that the association may be specific to difficulties identifying positive emotion only.

The affective prosodic recognition is closely related to positive symptoms in schizophrenia (see review of Alba-Ferarra et al. [42] ), and might be specific to those with auditory hallucination [22]. Dysfunctional superior temporal gyrus and disruption of the functional connectivity with anterior cingulate cortex may underlie the aberrant integration of prosodic features and misidentification of the source of the voice, which consequently contribute to hallucination formation [42]. Using dual-modality emotional stimuli, our results pinpoint a more specific correlation between failure of positive affective prosodic recognition and positive symptoms, suggesting that affective prosodic recognition of positive emotions can potentially be a specific neurocognitive measurement for auditory hallucination.

One of the limitations of the present study was the absence of an independent evaluation of concurrent mood status. Some might argue that failure of identifying happy emotion is secondary to depressive mood which is prevalent in patients of schizophrenia. In our data, the negative correlation between accuracy of happy emotion and positive symptoms remained significant after controlling single PANSS item score of depression (G4)(*r* = −0.27, *p*<0.01), or controlling the core Emotion factor score (G2,G3,G4,G6) of PANSS suggested by van der Gaag (2006) [43](*r* = −0.24, *p*<0.05). We also compared the emotion recognition ability between those who took antidepressant (N = 21) and those who did not (N = 89, see Table 3) but no significant difference has been found. Furthermore, adding either single depressive item or emotion factor into the stepwise regression model did not change the result (data not shown). Noteworthy, at least in our data, depression was not a significant mediating factor to the correlation between recognizing happy emotion and the existence of positive symptoms.

It was also a limitation without formal hearing and visual acuity test prior to the current study, since deficits within sensory modality may interfere ability to infer emotions of other people [21] which might further confound the results. Another limitation was the absence of a measurement of premorbid IQ. Though educational level could be a rough approximation of premorbid IQ, a more accurate measurement with proper matching between patients and healthy participants would be more convincing. However, as there is no consensus on how to measure premorbid IQ in a Han Chinese population this is a general limitation in studying this population. In addition, though the z-score calculation for neurocognitive performance of patients with schizophrenia were based on established data in Han Chinese community samples, the results would have been more convincing if the performance for the healthy controls were also acquired.

The association of deficits in positive emotion recognition and specific symptom domains suggests that deficits in emotional processing in schizophrenia are linked to the clinical expression of the disorder. Nonverbal emotion processing deficits exist across different sensory modalities in schizophrenia, but emotion and modality specific component of the deficits contributes to specific dimension cluster of clinical symptoms. In particular, less capable of identifying positive emotions lead to a more negative view of external world, and may also involves in the formation of positive symptoms. These relationships highlight the potential importance of deficits in emotional processing in the clinical expression of schizophrenia which may have mechanistic importance.
